# Comparison of the vitality test with sensitivity tests in mature and immature teeth: clinical trial

**DOI:** 10.1186/s12903-024-04317-3

**Published:** 2024-05-27

**Authors:** Funda Çağırır Dindaroğlu, Narin Özay Güngör

**Affiliations:** Faculty of Dentistry, Department of Pediatric Dentistry, İzmir Katip Çelebi University, İzmir, Turkey

**Keywords:** Pulp vitality, Pulse oximetry, Sensitivity test, Vitality test

## Abstract

**Background:**

One of the most important steps in deciding on the treatment of a tooth is to determine the vitality and health status of the pulp. Since immature teeth innervation is not completed, the response to sensitivity tests may not yield definite results. Pulse oximetry (PO) which is considered as a vitality test, measures the arterial oxygen saturation (SpO_2_). This study aims to compare PO, electric pulp test (EPT) and cold test in mature and immature permanent teeth.

**Methods:**

20 immature and 20 mature permanent incisors of 6-12-year-old ASA1 children who did not use any analgesics, were included in the study. Pulp vitality of the teeth was determined by EPT, cold test and PO. An infant probe of PO device (CMS60D, Contec Medical Systems Co. Ltd, China) was used to determine the SpO_2_ of the teeth. The SpO_2_ level is controlled on the patient’s finger by a children’s probe and an infant probe of PO. Shapiro-Wilk, Spearman rank correlation test and Kruskal-Wallis test/Dunn post-hoc analysis were used for statistical comparisons.

**Results:**

There was no significant correlation between finger SpO_2_ and the mature/immature teeth SpO_2_ (*r*=-0.026, *r* = 0.253). Arterial oxygen saturation values in the immature teeth were significantly higher than in the mature teeth (*p* = 0.002). There was a high correlation between the vitality response of the EPT, cold test and PO.

**Conclusions:**

Pulse oximetry can be used as an effective vitality test compared to sensitivity tests in both immature and mature permanent incisors.

## Introduction

One of the most important information necessary during the decision of the diagnosis of a tooth is determining the health status and vitality of the pulp [1]. The ideal method for determining pulpal health should be objective, noninvasive, easy to use clinically, reliable and noninvasive. In clinical practice, sensitivity tests and vitality tests are used to determine pulp vitality [2].

Sensitivity tests which evaluate the neuronal input, do not have the capability to show the exact blood flow and so they can only provide a piece of indirect information about the vitality of the tooth. The results of these tests are subjective, which can be verified with the sensitivity, pain threshold, fear and anxiety of the patients [3]. Electric pulp tests (EPT), thermal (cold and hot) tests, and cavity preparation tests were classified as sensitivity tests [4, 5].

The principle of the cold test is contraction and removal of the dentin liquid to the surface gives a stimulus to the A delta neurons in the dentin-pulp complex [6]. The most used cold tests are ice sticks (0 °C), dichlorodifluoromethane spray (-50 °C), ethyl chloride (-41 °C), and tetrafluoromethane spray (-26 °C) [7]. If there is no response to the cold test, the hot test can be applied. However, it is important to control the temperature to avoid permanent damage to the pulp tissue [8].

In the EPT, an electric current is applied to the tooth surface, and myelinated A-delta fibers within the pulp-dentin complex are stimulated. Local depolarization occurs in the fluid in the dentinal tubules, followed by an action potential occurs in the nerve fibers and a positive response is received from the EPT [9].

Pulse oximetry (PO) and laser Doppler, which are vitality tests, are non-invasive methods that evaluate the blood flow of the pulp [10, 11]. PO was developed in 1935 by Carl Matthes to measure oxygen-binding hemoglobin saturation values in tissues and used to determine arterial oxygen saturation (SpO_2_) values in arterial blood. It is used in clinical practice in many medical fields such as operating rooms, general anesthesia, sedation units, intensive care and endoscopy services. In the principle of PO, red (660 nm) and infrared (940 nm) lights are transmitted from one end of the sensor, while the photodetector on the other side of the tissue detects that oxygenated hemoglobin and oxygen-free hemoglobin absorbs different amounts of red and infrared light [12].

It was reported that sensitivity tests might give false positive or false negative results in traumatized and immature teeth [13]. As a result of trauma, nerve tissues in the pulp might remain active and false positive responses could be obtained [7]. Additionally, false negative responses could be obtained in immature primary and young permanent teeth due to immature innervations [14].

Although the pilot studies pointed out the limitations of PO as a pulp vitality test, many studies were conducted on a wide age range of patients reported the success of the PO [13, 15–18]. Dastmalchi et al. stated that PO was more reliable method than cold test, EPT and heat tests in the teeth that required endodontic treatment in 30-50-year-old patients [2]. Besides, PO was reported as a reliable and accurate vitality test than thermal tests for traumatized teeth [4] and also, it was shown that the PO had high sensitivity in determining the blood flow of anterior teeth in adolescents [19]. Sharma et al. found that PO and thermal tests were more accurate than EPT in primary and permanent teeth [20]. Although there are studies evaluated the PO as a vitality test, some of the confounding factors such as the wide age range of the sample, including different types of the teeth (incisors and molars), including the teeth with big restorations and a potential pulp inflammation restrict its use as a standard protocol in clinical use of the method.

This study aimed to compare the results of PO, EPT and cold test in permanent teeth with completed root development and incomplete permanent teeth. First null hypothesis of this study; there is no significant difference in vitality (SpO_2_) and sensitivity tests (EPT, cold test) results between mature and immature teeth. The second null hypothesis; there is no significant difference between EPT, PO and cold tests results.

## Methods

This parallel design, cross-sectional, controlled clinical study was completed in 2 months in İzmir Kâtip Çelebi University School of Dentistry, Department of Pediatric Dentistry.

### Ethics approval

This study was carried out in accordance with the Declaration of Helsinki and with the approval of the Izmir Kâtip Çelebi University Non-Interventional Clinical Research Ethics Committee (number: 0406). All the participants and their parents were informed about the study and signed an informed consent form.

### Sample selection

6–12 years old, ASA 1 dental pediatric patients between the age of 6 and 12 years who did not use any medication were included in the study. Patients with central incisors that had previous endodontic treatment or restorations larger than 2 mm and endodontic symptoms such as swelling, mobility, percussion and palpation sensitivity, spontaneous pain, dental trauma history, discoloration and sinus tract were excluded. Also, patients who did not have previously taken panoramic or periapical radiograph to determine the developmental stage of the apices of the central incisors were excluded.

### Clinical procedure

Vitality and sensitivity tests were performed by the same clinicians at İzmir Katip Çelebi University Faculty of Dentistry, Department of Pedodontics. Included central incisors were divided into two experimental groups based on their developmental stage as mature and immature. Besides, 10 endodontically treated central incisors from 10 children were included as a negative control group.

Group 1 (*n* = 23): Mature permanent central incisors.

Group 2 (*n* = 23): Immature permanent central incisors.

Group 3 (*n* = 10): Endodontically treated permanent central incisors (negative control).

Innervation of the teeth is associated with the developmental stage and age of the teeth. Therefore, we decided to include only central incisor teeth in 6-12-year-old pediatric patients to decrease the confounding factors to the comparison of the response to the vitality and sensibility tests of the teeth. To determine the vitality of teeth, an EPT (C PULSE pulp tester; Foshan COXO Medical Instruments Co Ltd, Foshan City, China), cold test (ice) and PO (CMS60D pulse oximeter Contec Medical Systems Co. Ltd, China) were used.

Before determining vitality using EPT, teeth were isolated with cotton rolls and suction and dried with an air spray. A piece of toothpaste was used as an electrolyte to ensure conductivity between the tooth surface and the electrode. For the measurements, the probe was placed on the incisal third of the crown where the pulp horns are close to the tooth surface. The threshold values at which the patient reacted to painful stimuli were recorded in the case forms.

**Fig. 1:**
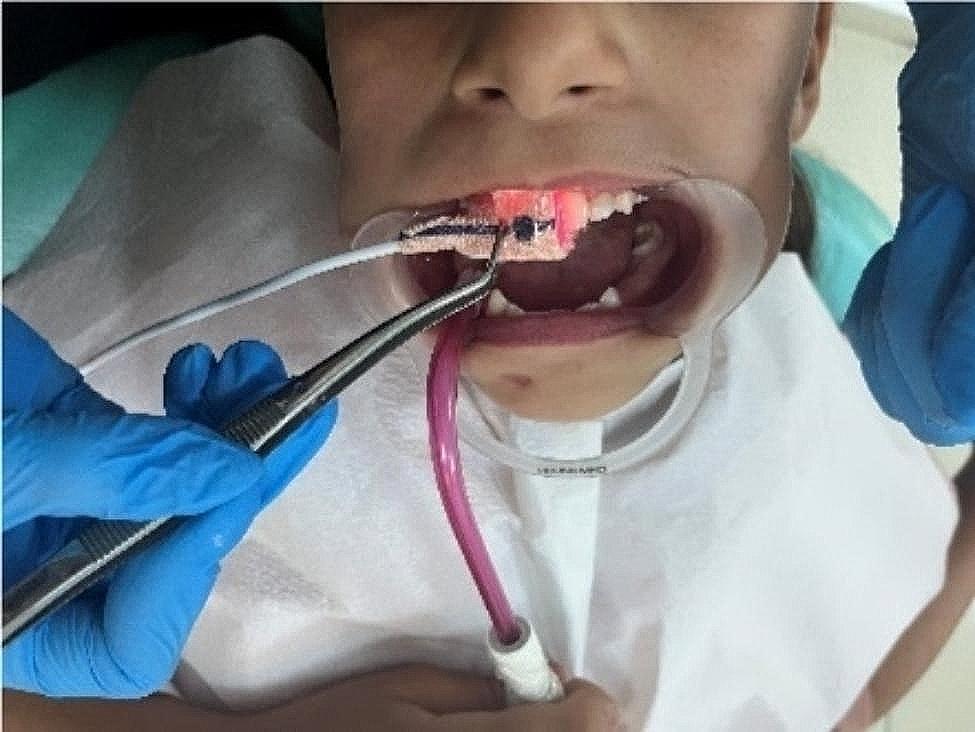
Pulse oximeter application on the incisor tooth

**Fig. 2:**
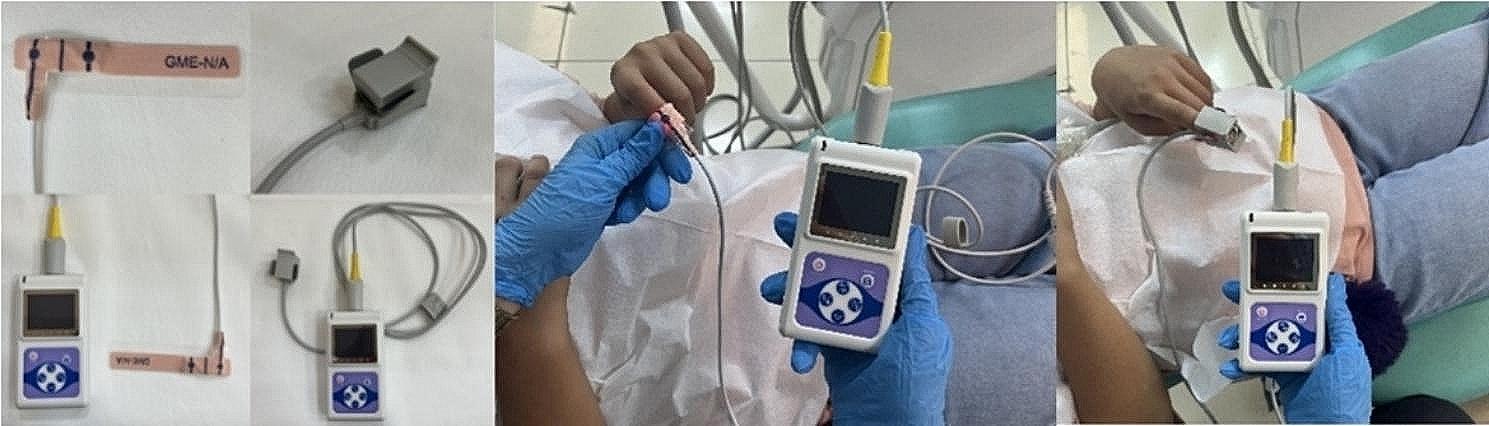
Measuring finger SpO_2_ value using PO infant probe and finger probe

The infant probe of the PO device was adapted to the tooth surface and the SpO_2_ level in the tooth was assessed. The sensors of the device were placed parallel and tightly on the buccal and palatal surfaces of the middle third of the tooth (Fig. 1). SpO_2_ level was also measured on the patient’s finger using the finger and infant probe of the PO (Fig. 2). Standardization was achieved by performing statistical calculations on the values of the right central incisors in all patients. The all measurement were performed 3 times and mean of the values were recorded.

Normal ice (0 °C) was used as a cold test method. A piece of the ice was placed on the incisal third of the permanent anterior teeth, waited until the patient felt pain, and the results were recorded.

### Sample size calculation

The sample size was calculated using G*Power software (3.1.9.7; Heinrich-Heine-Universität Düsseldorf, Düsseldorf, Germany) based on the parameter, SpO_2_ value. In a previously published study, the mean value was 86.71±2.052 for the immature teeth and 84.61±2.170 for the mature teeth [15]. To test the null hypothesis that there was no difference in SpO_2_ level between the groups, it was determined that a sample size of 23 in each group reached 95% power on a significance level of *p*<0.05.

### Statistical analysis

Statistical analysis was performed with SPSS Statistics 25 (IBM, Armonk, New York). Descriptive statistics were calculated for each experimental group and negative control group. Shapiro-Wilk normality test was used to analyze the distribution of the measurements. Correlations between SpO_2_ measurements from mature-immature tooth and finger (using infant probe and finger probe) were performed using the Spearman correlation coefficient. The comparison of SpO_2_ and EPT measurements based on the tooth development status was made using the Kruskal-Wallis test/Dunn post-hoc analysis. *p* < 0.05 was considered significant.

## Results

Twenty-three mature and 23 immature permanent central incisors were included in the study from 46 pediatric patients. Vitality and sensitivity test results of mature and immature teeth are shown in Table 1. In the negative control group, the SpO_2_ value was 74% in only one tooth and 0 on the other 9 teeth. All of the mature and immature teeth in the experimental groups showed positive responses and all of the endodontically treated incisors in the negative control group showed negative responses to the EPT and cold test (Table 1).

**Table 1:** Results of vitality and sensitivity tests

Dental development status	Testing methods					
	Electric pulp test		Cold test		Measured from teeth (infant probe)	
	Positive	Negative	Positive	Negative	Positive	Negative
Mature teeth (n)	46	0	46	0	46	0
Immature teeth (n)	46	0	46	0	46	0
Endodontically treated teeth -Negative Control (n)	0	10	0	10	1	9

The mean SpO_2_ values were 98.18% from fingers, 86.65% (minimum: 81, maximum: 92) from immature incisors and 82.65% (minimum: 80, maximum: 85) from mature incisors. Arterial oxygen saturation values measured in mature and immature teeth were lower than the values measured from the finger in both groups. The median SpO_2_ value was significantly lower in mature teeth compared to immature teeth (*p* < 0.05). In the negative control group mean and median SpO_2_ values were 7.40 and 0, respectively (Table 2).

**Table 2:** SpO_2_ descriptive values based on root development status

	Mean SpO_2_	Median SpO_2_	Standard deviation	Minimum	Maximum
Mature teeth	82.65	83	1.58	80	85
Immature teeth	86.65	86	3.27	81	92
Endodontically treated teeth -Negative Control	7.40	0	23.4	0	74

There was no correlation between finger SpO_2_ (measured using infant probe and finger probe) and mature/immature teeth SpO_2_ (*r*=-0.026, *r*=-0.119, *r* = 0.253, *r* = 0.357, respectively). There was no correlation between the values measured from the finger in the measurements made in the control group with root canal treatment (*r*=-0.192, *r*=-0.192). There were high correlation in the finger SpO_2_ values between using the infant and the finger probe (*r* = 0.751), (Table 3).

**Table 3:** Correlations between SpO_2_ values measured from teeth and fingers according to tooth developmental status

	Mature teeth SpO_2_ (infant probe)	Immature teeth SpO_2_ (infant probe)	Endodontically treated teeth-Negative Control	SpO_2_ Measured from finger (infant probe)	SpO_2_ Measured from finger (finger probe)
Mature teeth SpO_2_ (infant probe)	1	-	-	-0.026	-0.119
Immature teeth SpO_2_ (infant probe)	-	1	-	0.253	0.357
Endodontically treated teeth-Negative Control	-	-	1	-0.192	-0.192
SpO_2_ Measured from finger (infant probe)	-0.026	0.253	-0.192	1	0.751
SpO_2_ Measured from finger (finger probe)	-0.119	-	-	0.751	1

Spearman’s correlation analysis

Pairwise comparisons of mature teeth SpO_2_, immature teeth SpO_2_ and negative control teeth SpO_2_ were shown in Table 4. A significant difference was found between the mean difference of mature teeth SpO_2_ and immature teeth SpO_2_ (*p* = 0.002). The mean of immature teeth SpO_2_ was statistically significantly higher than mature teeth SpO_2_. The mean differences in endodontically treated control group and mature/immature teeth were statistically significant (*p* = 0.004, *p* < 0.001, respectively) (Table 4).

**Table 4:** Comparison of SpO_2_ values based on dental developmental status and negative control group

	Tooth development status	Tooth development status	Mean difference	Standard error	*p*	95% confidence interval	
						Lower bound	Upper bound
SpO_2_	Mature teeth	Immature teeth	-4	4.782	0.002*	-5.9	-2.09
		Endodontically treated teeth-Negative Control	75.2	6.143	0.004*	53.6	96.8
	Endodontically treated teeth-Negative Control	Mature teeth	-75.2	6.143	0.004*	-96.8	-53.6
		Immature teeth	-79.2	6.143	< 0.001*	-100.8	-57.6

Kruskal Wallis test; Dunn’s post-hoc test

* Significant *p* value at 0.05 level

The median values of EPT measurements were found to be 21.30 s (minimum: 11 s, maximum: 33 s) in mature teeth, 44.43 s (minimum: 30 s, maximum: 61 s) in immature teeth, and 0 s in endodontically treated-negative control group. The EPT value in immature teeth was found to be significantly higher compared to mature teeth (*p* < 0.001). In the comparison of the EPT values of the immature- mature teeth and the negative control teeth, there was a significant difference between the 3 groups (Table 5) (*p* < 0.001).

**Table 5:** Comparison of EPT values based on tooth developmental status

	Tooth development status	Tooth development status	Mean difference	Standard error	*p*	95% confidence interval	
						Lower bound	Upper bound
EPT-Second	Mature teeth	Immature teeth	-23.1	4.794	< 0.001*	-28.81	-17.45
		Endodontically treated teeth-Negative Control	21.3	6.159	0.021*	18.02	24.59
	Immature teeth	Mature teeth	23.1	4.794	< 0.001*	17.45	28.81
		Endodontically treated teeth -Negative Control	44.4	6.159	< 0.001*	39.56	49.31
	Endodontically treated teeth -Negative Control	Mature teeth	-21.3	6.159	0.021*	-24.59	-18.02
		Immature teeth	-44.4	6.159	< 0.001*	-49.31	-39.56

Kruskal Wallis test; Dunn’s post-hoc test * Significant *p* value at 0.05 level

EPT: Electric pulp test

## Discussion

Electric pulp test in immature teeth may not be reliable because of incomplete innervation and blood supply is a factor that helps healing in immature teeth. So determining SpO_2_ values is important for correct treatment planning. As a result of this study, the PO can be used safely in both mature and immature teeth, and it showed comparable results to sensitivity tests such as EPT and cold test. Therefore, both null hypotheses were accepted in this study.

There are different suggestions in the literature on clinical use regarding how long the measurement should take to determine SpO_2_ using PO in anterior permanent teeth. In a previous study, it was recommended that measurements should be made in 20–30 s in order to reduce artifacts [16]. Similar studies reported that the probe’s application time can vary between 5 and 45 s, regardless of the tooth type [17, 18]. Considering the previously published studies, the SpO_2_ value on the screen was recorded 30 s after the application of the probes on the teeth in this study.

In this study, SpO_2_ values measured from mature and immature teeth were lower than those measured from the patients’ fingers similar to the previous studies [15, 19]. There was no correlation between SpO_2_ values measured from fingers and teeth in all 3 groups. Similarly, in a study conducted on mature and immature teeth of children aged 4–12 years, there was no correlation between the results measured from the finger and the tooth [20].

Arterial oxygen saturation values in immature teeth were significantly higher compared to mature teeth. Since only one measurement of SpO_2_ was higher than 0 in the negative control group, a highly significant difference was found between the immature and negative control groups. The mean difference between the immature teeth and negative controls was higher compared to the difference between mature and immature teeth. There was a significant difference between the stage of root development based on SpO_2_ of the teeth. Similar results were obtained in the study conducted by Bargrizan et al. [15]. This result is consistent with the fact that the blood supply decreases as the root development progresses and the SpO_2_ value decreases concomitantly. In the same study, it was reported that the accuracy rate of thermal pulp test and PO were 100% [15]. In the present study, PO showed 74% SpO_2_ value in 1 tooth with root canal treatment in the negative control group. However, a tooth that showed 77% or less SpO_2_ rate, is considered devital [17]. Therefore, the accuracy rate of the PO was 100% in this study. And also, SpO_2_ values in mature and immature vital teeth were between 80 and 92%.

Karayılmaz et al. reported that the PO device was insufficient to determine vitality of devital teeth due to the light reflection from the restoration material spreading to the gingiva and resulted in a false positive response, and also PO was sensitive to clinical practice. Therefore, they did not recommend the PO in teeth with large restorations near to the gingiva [19]. In our study, restorations larger than 2 mm were excluded from the study.

The position where the sensors of the PO are placed on the tooth surface is important for accurate results. It was recommended that the sensors of the PO device should tightly touch the tooth surface compatible with the dental anatomy and should be located parallel to each other [21]. In this study, the infant sensors of the PO device were placed parallel to each other on the labial and palatal surfaces.

The accuracy rate of EPT was reported as 90% for permanent teeth [15]. In the present study, there was no response in the control group so there was no any false positive response. Therefore, the accuracy rate of the cold and EPT was 100%. We suggest that the inconsistence was associated with the difference in the used device, the age of the samples and the teeth.

Similar to the positioning of the PO device on the tooth surface, the position in which the EPT device is placed on the tooth is also important for the accuracy of the results. In permanent teeth, neural structures are mostly located in areas close to the pulp horns [22]. In the anterior teeth, the pulp horns are close to the incisal third and the enamel thickness is low in that area, so the EPT sensor was placed near the incisal third of the labial surface. Bender et al. reported that the threshold value decreased due to the application of EPT on the incisal third region of the anterior teeth caused a decreased threshold value, and the results were 100% accurate [23]. Since the incisal third of the crown was reported as the best option, we placed the probe on the same area. In a previous study, using toothpaste as an electrolyte can provide high voltage readings in EPT measurement, and all different kinds of toothpaste conducted voltage sufficiently [24]. Therefore, we decided to use toothpaste as a conducting media.

Determination of vitality is critical for the survival of the teeth. Because of crack risk in root canal treatment, unnecessary root canal treatment can result in tooth loss [25].

Although we determined the sample size using a power analysis taking the results of a previous study into consideration; the necessary sample size for different assumptions may be different. In this regard, the sample size of this study can be considered small which can be a possible limitation of the study [15]. In addition, being a single-center study limits the diversity of the study population and can be stated as another limitation. In this study, the measurements were repeated 3 times to avoid measurement errors. Because of the cross-sectional design of the study, follow-up sessions were not planned. Further study with follow-up sessions to evaluate the change in vitality test response through the developmental stage can contribute to the literature. Furthermore, PO was compared with the sensitivity test which can be affected by the pain threshold and anxiety of the patients. One more vitality test such as Doppler flowmetry could be added to the comparisons. Finally, the results of this study reflect the clinical diagnosis of anterior teeth and are not applicable to posterior teeth. The readers should consider this in their clinical practice.

The response to the sensitivity tests depends on tooth maturity, patients’ age and cooperation. Besides the reliability of the tests may be affected because of the painful stimulus and false negative or false positive results may be obtained in uncooperative pediatric patients [15]. Therefore, more objective alternative tests may be required to assess the vitality of teeth in pediatric patients. Pulse oximetry is an atraumatic, noninvasive, objective, and painless approach, it can be easily used in children who have dental fear and anxiety [15, 26]. In this study, there was no children had any cooperation problems as a result of the use of the PO.

## Conclusions

Although sensitivity tests were suggested that there are some limitation to use them to determine pulp vitality in immature teeth in previous studies, their accuracy was %100 in this study. With a similar accuracy rate, pulse oximetry can be considered as an alternative vitality test to existing sensitivity tests to determine pulp vitality for mature and immature teeth, especially in pediatric dental patients.

## Data Availability

The datasets used and analysed during the current study available from the corresponding author on reasonable request.

## Abbreviations

EPT: Electric pulp tests
PO: Pulse oximetry
SpO_2_: Arterial oxygen saturation
